# Contrastive Learning-Based Personalized Tag Recommendation

**DOI:** 10.3390/s24186061

**Published:** 2024-09-19

**Authors:** Aoran Zhang, Yonghong Yu, Shenglong Li, Rong Gao, Li Zhang, Shang Gao

**Affiliations:** 1 School of Computer Science, Jiangsu University of Science and Technology, Zhenjiang 212000, China; justzhangaoran@gmail.com (A.Z.); gao_shang@just.edu.cn (S.G.); 2 College of Tongda, Nanjing University of Posts and Telecommunication, Yangzhou 225127, China; nsqaq9898@163.com; 3 School of Computer Science, Hubei University of Technology, Wuhan 430068, China; gaorong@hbut.edu.cn; 4 Department of Computer Science, Royal Holloway University of London, Egham TW20 0EX, UK; li.zhang@rhul.ac.uk

**Keywords:** contrastive learning, graph neural network, personalized tag recommendation

## Abstract

Personalized tag recommendation algorithms generate personalized tag lists for users by learning the tagging preferences of users. Traditional personalized tag recommendation systems are limited by the problem of data sparsity, making the personalized tag recommendation models unable to accurately learn the embeddings of users, items, and tags. To address this issue, we propose a contrastive learning-based personalized tag recommendation algorithm, namely CLPTR. Specifically, CLPTR generates augmented views of user–tag and item–tag interaction graphs by injecting noises into implicit feature representations rather than dropping nodes and edges. Hence, CLPTR is able to greatly preserve the underlying semantics of the original user–tag or the item–tag interaction graphs and avoid destroying their structural information. In addition, we integrate the contrastive learning module into a graph neural network-based personalized tag recommendation model, which enables the model to extract self-supervised signals from user–tag and item–tag interaction graphs. We conduct extensive experiments on real-world datasets, and the experimental results demonstrate the state-of-the-art performance of our proposed CLPTR compared with traditional personalized tag recommendation models.

## 1. Introduction

As an important subfield of recommendation systems [1,2,3], personalized tag recommendation (i.e., PTR) systems [4,5,6,7,8], has become increasingly popular in both academia and industry. By modeling historical behaviors among entities, the PTR system generates a personalized tag list for each user. These tags are very useful for users to manage and retrieve items. Classic PTR algorithms include HOSVD [4], RTF [5], and PITF [6]. Although classic PTR algorithms model the third-order interaction relationships among entities within a unified framework, they ignore high-order collaborative signals among users, items, and tags, which is harmful to the expressive capacity of PTR models. Recently, the deep learning technique has been widely used in computer vision [9,10,11] and natural language processing [12,13,14]. Meanwhile, some researchers [15,16,17] utilize Graph Convolutional Neural Networks (i.e., GCNs [18]) to capture high-order collaborative signals among entities and propose some GCN-based PTR models. Essentially, these methods capture the similarities among high-order entities through GCN, which improves the performance of PTR models. Although these works have shown that integrating GCN into PTR models is a promising approach, GCN-based PTR models are still limited by data sparsity.

**Figure 1:**
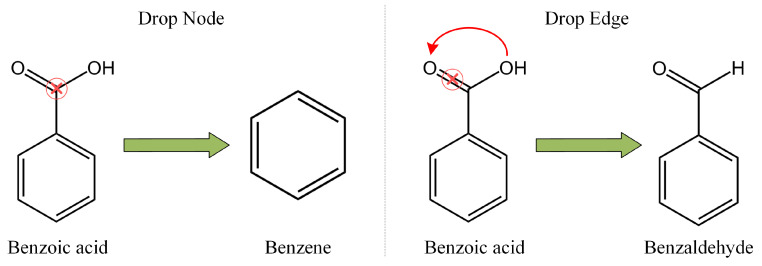
A toy example of random augmentations for benzoic acid.

For recommendation systems, the contrastive learning technique [19,20,21] is an effective solution to alleviate the problem of data sparsity. Most contrastive learning-based item recommendation models adopt the principle of InfoMax [20,22,23], which maximizes the consistency between positive pairs and pulls them closer while minimizing the consistency between negative pairs and pushing them away. However, they usually adopt random augmentation strategies (i.e., drop node, drop edge) to obtain two augmented views, which is unable to guarantee that the two augmentations are positively correlated. As shown in Figure 1, benzoic acid is a common food preservative. If we drop a carbon atom from benzoic acid by dropping operation, benzoic acid will turn into benzene. However, benzene is a highly toxic chemical substance with completely different chemical properties compared to those of benzoid acid. In addition, if we break the chemical bond between oxygen and carbon atoms in benzoic acid through dropping edge operation, benzoic acid will transform into benzaldehide. Unlike benzoid acid, benzaldehide is widely utilized in food seasoning. Therefore, adopting random augmentation strategies to obtain augmented views will change the structural information of the original graphs.

**Figure 2:**
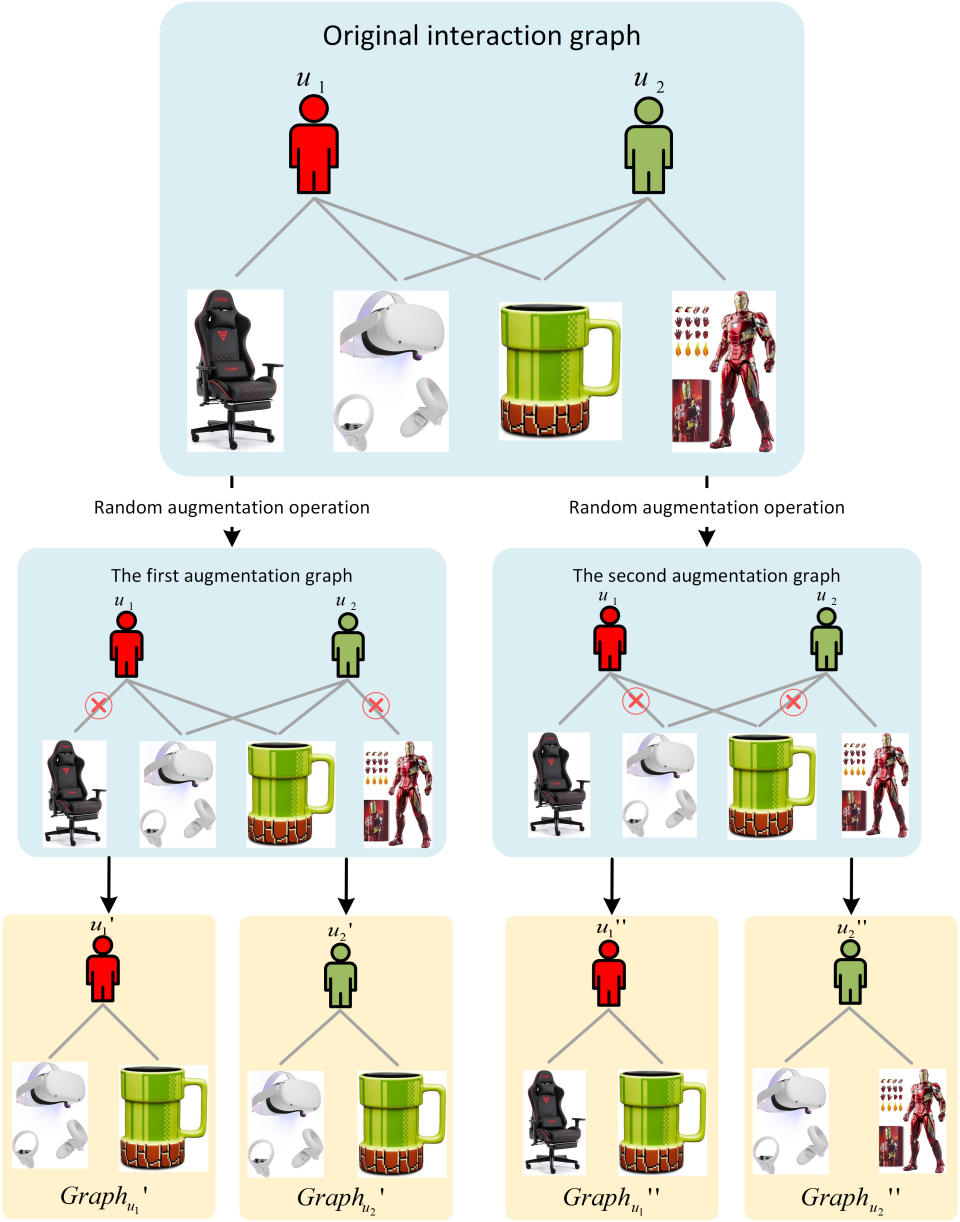
An example of random augmentations for user–item interaction graph.

For personalized item recommendation, random augmentation strategies would change the underlying semantics of the original user–item bipartite graph. Moreover, random augmentation strategies may also introduce false negative samples. For instance, assume user $u_{1}$ has purchased items $I_{1}=\left\{Chair,VR,Mug\right\}$ and user $u_{2}$ has purchased items $I_{2}=\left\{VR,Mug,Toy\right\}$. According to the historical records of users $u_{1}$ and $u_{2}$, the original graph is presented in Figure 2. By utilizing dropping edge operation, we can obtain the first augmented view, i.e., $Graph_{u_{1}}^{\prime }$ and $Graph_{u_{2}}^{\prime }$, and the second augmented view, i.e., $Graph_{u_{1}}^{\prime \prime }$ and $Graph_{u_{2}}^{\prime \prime }$. According to the principle of contrastive learning, $u_{1}^{\prime }$ and $u_{1}^{\prime \prime }$ should be a positive pair. Meanwhile, $u_{1}^{\prime }$ and $u_{2}^{\prime }$ should be a negative pair. However, $u_{1}^{\prime }$ and $u_{2}^{\prime }$ share common preferences since they visit the same set of items. Hence, $u_{1}^{\prime }$ and $u_{2}^{\prime }$ is a false negative pair. In general, the random augmentation may introduce false negative samples, resulting in the recommendation models unable to accurately distinguish positive and negative pairs.

To tackle the above issues, we propose a contrastive learning-based personalized tag recommendation model, namely CLPTR. Specifically, we inject noises into the embeddings of users, items, user-specific tags, and item-specific tags to generate augmented views. In this way, we preserve the underlying semantic structures of original user–tag and item–tag interaction graphs and avoid the problem of false negatives caused by inappropriate augmentation strategies. In addition, we integrate the contrastive learning module into PTR and learn the embeddings of entities by maximizing the consistency between augmented views. We summarizes the main contributions of this paper as follows:

- We utilize a noise augmentation strategy to generate augmented views of user–tag and item–tag interaction graphs, which effectively guarantees that the underlying semantics of original interaction graphs remain unchanged and avoids the problem of false negatives.
- We integrate the contrastive learning module into PTR, which is able to effectively alleviate the problem of data sparsity.
- We conduct extensive experiments on real-world datasets, and the experimental results demonstrate the superior performance of our proposed CLPTR compared with traditional PTR models.

## 2. Related Work

### 2.1. Personalized Tag Recommendation Algorithms

Classic PTR algorithms include HOSVD [4], RTF [5] and PITF [6]. For example, HOSVD [4] utilizes a high-order singular value decomposition technique to factorize the third-order tensor of the user–item tags and captures the latent semantic associations among entities. However, HOSVD focuses on the user ratings for tags and ignores the rankings between tags. To tackle this issue, Rendle et al. [5] proposed ranking with tensor factorization. But HOSVD and RTF are unable to scale to large datasets due to their high computational complexity. In order to reduce the computational cost, Rendle et al. [6] extended the BPR criteria to the PTR and proposed PITF. In addition, Fang et al. [7] proposed NLTF, which utilizes Gaussian kernels to enhance the capacity of modeling the complex relations among entities. Although the above works model underlying semantic relationships among entities by tensor decomposition, they only consider first-order collaborative signals and ignore the higher-order collaborative signals. Recently, some researchers utilized deep learning techniques to boost the performance of PTR models. For instance, Yuan et al. [8] proposed ABNT. ABNT employed multi-layer perceptron to capture the nonlinear relationships among entities. In addition, Chen et al. [16] proposed GNN-PTR, which integrates GCN into PTR models. To reduce the training difficulty of the PTR model, Yu et al. [17] proposed LNGTR, which is a lightweight variant of GNN-PTR. However, GCN-based PTR models still suffer from data sparsity.

### 2.2. Contrastive Learning-Based Recommendation Models

Contrastive learning is able to extract self-supervised signals from raw data, effectively reducing the problem of data sparsity in recommendation systems. For personalized item recommendations, Wu et al. [19] proposed self-supervised graph learning for recommendation, namely SGL, which integrates the contrastive learning module into a personalized item recommendation model. In addition, SGL utilizes a random augmentation strategy to generate augmented views and employs InfoNCE to learn the embeddings of nodes of the user–item bipartite graph. However, it is necessary for SGL to reconstruct the adjacency matrix in each training epoch, which is time consuming. To address this issue, Yu et al. [21] proposed SimGCL. SimGCL utilizes the noise augmentation strategy to improve the efficiency of model training. In order to reduce the popularity bias, Liu et al. [24] proposed a popularity-aware debiased contrastive loss for collaborative filtering. For sequential recommendation, Xie et al. [25] proposed contrastive learning for sequential recommendation, namely CL4SRec, which integrates the contrastive learning module into the transformer-based sequential recommendation model. Moreover, CoSeRec [26] utilizes substitute and insert operations to generate robust augmented sequences. Chen et al. [27] proposed intent contrastive learning for sequential recommendation, employing clustering methods to learn the potential intentions of users in item sequences, and injecting the user intentions into contrastive learning-based sequential recommendation. For tag-enhanced item recommendation, Wu et al. [28] proposed intent-aware multi-source contrastive alignment for item recommendation, namely IMCAT. IMCAT utilizes a user intent-aware self-supervised pairing process to make the pairing process more fine-grained and avoid embedding collapse. Moreover, Xu et al. [29] proposed tag-aware graph contrastive learning, namely TAGCL. TAGCL jointly optimizes TAGCL through negative tag loss and TransT regularization, which helps the model learn high-quality features. Although most existing works enhance the performance of recommendation models to a certain extent, they destroy the underlying semantic relationship of user–item interaction information and introduce false negative samples, since they utilize random augmentation strategies to obtain augmented views. Moreover, the contrastive learning technique has not been explored in the PTR systems. In this paper, we integrate the contrastive learning module into the PTR model and alleviate the problem of data sparsity.

## 3. Contrastive Learning-Based Personalized Tag Recommendation

The contrastive learning technique is a promising approach to reduce the problem of data sparsity and is investigated by other recommendations, such as item recommendations and sequential recommendations. For item recommendation, SGL [19] and SimGCL [21] utilize contrastive learning to improve the performance of models. For sequential recommendation, representative contrastive learning-enhanced methods include CL4SRec [25] and CoSeRec [26]. These above classical methods demonstrate that the contrastive learning technique is a promising approach to improve the performance of recommendation models.

**Figure 3:**
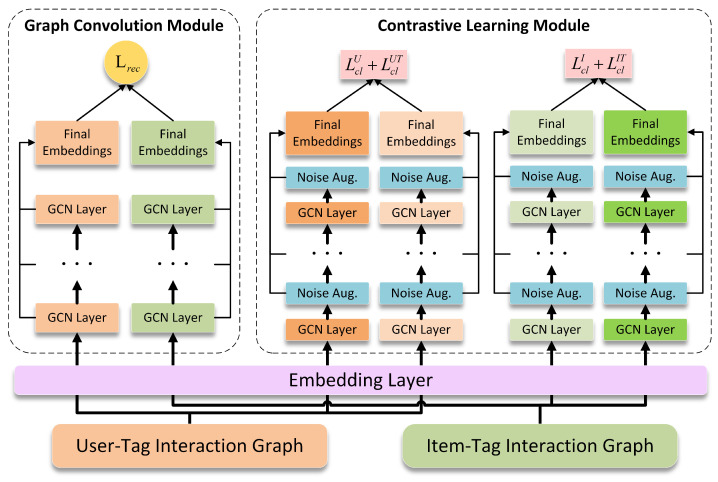
The framework of the contrastive learning-based personalized tag recommendation model.

In this section, we introduce the proposed contrastive learning-based personalized tag recommendation model, which is called CLPTR. CLPTR mainly consists of two important components, i.e., a graph convolution module and a contrastive learning module. We utilize the graph convolution module to capture the collaborative signals and inject the collaborative signals into the embeddings of entities. In addition, the contrastive learning module generates augmented views of user–tag and item–tag interaction graphs by injecting noises into implicit feature representations rather than dropping nodes and edges. Moreover, the contrastive learning module utilizes InfoNCE to learn the embeddings of entities by pulling the embeddings of positive pairs close and pushing the embeddings of negative pairs away. The framework of the contrastive learning-based personalized tag recommendation model is illustrated in Figure 3.

### 3.1. Problem Description

In this article, we focus on PTR tasks. The PTR systems typically include three entities: the set of users $U=\{u_{1},u_{2},\cdots ,u_{\left|U\right|}\}$, the set of items $I=\{i_{1},i_{2},\cdots ,i_{\left|I\right|}\}$ and the set of tags $T=\{t_{1},t_{2},\cdots ,t_{\left|T\right|}\}$. We utilize S⊆U×I×T to denote the users’ historical tagging behaviors. A ternary (u,i,t)∈S indicates that the item *i* is annotated with the tag *t* by user *u*. The main purpose of PTR systems is to compute the probability of the user *u* annotating tags to item *i* and recommend the top-N tags with the highest probabilities to user *u*.

### 3.2. Graph Convolution Module

The graph convolution module is mainly utilized to capture high-order collaborative signals among entities and enrich the semantic information of the representations of entities. Each component of the graph convolution module is described in the following sections.

#### 3.2.1. Embedding Layer

Given a triplet (u,i,j), we map entities (i.e., users, items, and tags) to a low-dimensional embedding space based on their ID, formally,

(1) $$\begin{array}{c} e_{u}^{\left(0\right)}=lookup\left(U,u\right),\;e_{i}^{\left(0\right)}=lookup\left(I,i\right),\\ e_{ut}^{\left(0\right)}=lookup\left(T_{U},t\right),\;e_{it}^{\left(0\right)}=lookup\left(T_{I},t\right), \end{array}$$

where the lookup(·) operation retrieves the latent feature vector from the embedding matrix based on the ID of the entity. $U\in R^{|U|\times K}$ is the latent user feature matrix, $I\in R^{|I|\times K}$ is the latent item feature matrix, $T_{U}\in R^{|T|\times K}$ is the latent user-specific tag feature matrix and $T_{I}\in R^{|T|\times K}$ is the latent item-specific tag feature matrix. *K* denotes the embedding size.

#### 3.2.2. Embedding Propagation Layer

PTR systems include user–item, user–tag and item–tag interaction graphs. However, the user–item interaction will vanish for predicting rankings for Bayesian Personalized Ranking optimization. Hence, similar to PITF, GNN-PTR, and LNGTR, we only take the user–tag and item–tag interaction graphs into account. The main purpose of the embedding propagation layer is to utilize the message-passing mechanism to capture high-order collaborative signals among entities on the interaction graphs. Taking the user–tag interaction graph as an example, we employ the lightweight GCN model to capture the high-order collaborative signals. At the $l^{th}$ layer, the embeddings of user $e_{u}^{\left(l\right)}$ and user-specific tag $e_{ut}^{\left(l\right)}$ are formulated as follows

(2) $$e_{u}^{\left(l\right)}=Agg_{u}\left(\left\{e_{ut}^{\left(l-1\right)},\;\forall t\in N_{u}\right\}\right),e_{ut}^{\left(l\right)}=Agg_{ut}\left(\left\{e_{ut}^{\left(l-1\right)},\;\forall u\in N_{t}\right\}\right),$$

where $e_{u}^{\left(l-1\right)}$ and $e_{ut}^{\left(l-1\right)}$ indicate the embeddings of user *u* and user-specific tag *t* at the ${\left(l-1\right)}^{th}$ embedding propagation layer, respectively. $Agg_{u}\left(\cdot \right)$ and $Agg_{ut}\left(\cdot \right)$ are the user aggregation function and user-specific tag aggregation function, respectively. $N_{u}$ is the set of tags that connect to user *u* in the user–tag interaction graph, while $N_{t}$ is the set of users that interacts with tag *t*. Similar to LightGCN [30], the weight sum strategy is utilized to compute the user and user-specific tag aggregation functions, formally,

(3) $$e_{u}^{\left(l\right)}=\sum_{t\in N_{u}}\frac{1}{\sqrt{|N_{u}|}\sqrt{|N_{t}|}}e_{ut}^{\left(l-1\right)},\;e_{ut}^{\left(l\right)}=\sum_{u\in N_{t}}\frac{1}{\sqrt{|N_{u}|}\sqrt{|N_{t}|}}e_{u}^{\left(l-1\right)}.$$

According to Equation (3), $e_{u}^{\left(1\right)}$ and $e_{ut}^{\left(1\right)}$ capture the one-hop connectivity information by aggregating direct neighbor information. By recursively stacking more embedding propagation layers, we are able to inject the high-order collaborative signals into the embeddings of users and tags. Similarly, on item–tag interactions graph, we also recursively stack the embedding propagation layer to capture the high-order collaborative signals between items and tags.

#### 3.2.3. Prediction Layer

By stacking *L* embedding propagation layers, we obtain the sets of embeddings of entities:

(4) $$\begin{array}{c} \left\{e_{u}^{\left(1\right)},e_{u}^{\left(2\right)},\cdots ,e_{u}^{\left(L\right)}\right\},\left\{e_{i}^{\left(1\right)},e_{i}^{\left(2\right)},\cdots ,e_{i}^{\left(L\right)}\right\},\\ \left\{e_{ut}^{\left(1\right)},e_{ut}^{\left(2\right)},\cdots ,e_{ut}^{\left(L\right)}\right\},\left\{e_{it}^{\left(1\right)},e_{it}^{\left(2\right)},\cdots ,e_{it}^{\left(L\right)}\right\}, \end{array}$$

Each element of one set describes the various aspects of entity characteristics. We obtain the final representations of each entity by combining all corresponding elements to simultaneously encode both low-order and high-order collaborative signals, formally,

(5) $$\begin{array}{c} e_{u}^{*}=\sum_{l=0}^{L}\alpha_{l}\cdot e_{u}^{\left(l\right)},e_{i}^{*}=\sum_{l=0}^{L}\alpha_{l}\cdot e_{i}^{\left(l\right)},\\ e_{ut}^{*}=\sum_{l=0}^{L}\alpha_{l}\cdot e_{ut}^{\left(l\right)},e_{it}^{*}=\sum_{l=0}^{L}\alpha_{l}\cdot e_{it}^{\left(l\right)}, \end{array}$$

where $\alpha_{l}=\frac{1}{L+1}$ is the weight coefficient for the embedding of entities obtained at the $l^{th}$ layer. In this way, we enrich the semantics of entities involved in three-order interaction relationships. Finally, given the final representations of user *u*, item *i*, and tag *t*, we predict the score of user *u* annotating item *i* with the tag *t* as follows,

(6) $${\hat{p}}_{u,i,t}=e_{u}^{*}⊙e_{ut}^{*}+e_{i}^{*}⊙e_{it}^{*},$$

where ⊙ denotes the dot product operation.

### 3.3. Contrastive Learning Module

The contrastive learning module firstly generates augmented views of user–tag and item–tag interaction graphs by injecting noises into implicit feature representations. Then, the contrastive learning module utilizes InfoNCE to learn the embeddings of entities by pulling the embeddings of positive pairs close and pushing the embeddings of negative pairs away.

#### 3.3.1. Noise Augmentation

In order not to destroy the original structural relationships among interaction graphs and introduce false negatives, CLPTR injects noises into the implicit feature representations to obtain augmented views. On the user–tag interaction graph, the embeddings of user and user-specific tag via two noise augmentation operations are as follows:

(7) $$\begin{array}{c} {e_{u}^{*}}^{\prime }=\sum_{l=0}^{L}\alpha_{l}\cdot \left[e_{u}^{\left(l\right)}+{\left(\Delta_{u}^{\left(l\right)}\right)}^{\prime }\right],\\ {e_{u}^{*}}^{\prime \prime }=\sum_{l=0}^{L}\alpha_{l}\cdot \left[e_{u}^{\left(l\right)}+{\left(\Delta_{u}^{\left(l\right)}\right)}^{\prime \prime }\right],\\ {e_{ut}^{*}}^{\prime }=\sum_{l=0}^{L}\alpha_{l}\cdot \left[e_{ut}^{\left(l\right)}+{\left(\Delta_{ut}^{\left(l\right)}\right)}^{\prime }\right],\\ {e_{ut}^{*}}^{\prime \prime }=\sum_{l=0}^{L}\alpha_{l}\cdot \left[e_{ut}^{\left(l\right)}+{\left(\Delta_{ut}^{\left(l\right)}\right)}^{\prime \prime }\right], \end{array}$$

where ${\left(\Delta_{u}^{\left(l\right)}\right)}^{\prime }$, ${\left(\Delta_{u}^{\left(l\right)}\right)}^{\prime \prime }$, ${\left(\Delta_{ut}^{\left(l\right)}\right)}^{\prime }$, and ${\left(\Delta_{ut}^{\left(l\right)}\right)}^{\prime \prime }$ are noise vectors, and $\left|\right|\Delta^{\left(l\right)}{\left|\right|}_{2}=\varepsilon$, $\Delta^{\left(l\right)}={\bar{\Delta }}^{\left(l\right)}⊙sign\left(e_{*}^{\left(l\right)}\right)$. ε controls the magnitude of noise $\Delta^{\left(l\right)}$, and $\Delta^{\left(l\right)}$ is regarded as a point on a hypersphere with radius ε. ${\bar{\Delta }}^{\left(l\right)}$ follows a uniform distribution, i.e., ${\bar{\Delta }}^{\left(l\right)}∼U\left(0,1\right)$. In addition, for the item–tag interaction graph, we utilize similar operations to generate the embeddings of items and items-specific tags, i.e., ${e_{i}^{*}}^{\prime }$, ${e_{i}^{*}}^{\prime \prime }$, ${e_{it}^{*}}^{\prime }$, ${e_{it}^{*}}^{\prime \prime }$. In fact, the process of noise augmentation is to rotate small angles of the original feature vectors. This augmentation scheme is able to effectively preserve the original semantics of entities and keep the structure of user–tag and item–tag interaction graphs unchanged.

#### 3.3.2. Contrastive Loss

We utilize InfoNCE to calculate the contrastive loss of CLPTR. Specifically, we treat two augmented views derived from the same node as positive pairs and other nodes as negative pairs. InfoNCE maximizes the similarities among the positive pairs and minimizes the similarities among negative pairs. For the user–tag interaction graph, we calculate the contrastive loss of user side $L_{cl}^{U}$ and the contrastive loss of tag side $L_{cl}^{UT}$ as follows,

(8) $$\begin{array}{c} L_{cl}^{U}=\sum_{u\in U}-\log\frac{\exp(sim\left({e_{u}^{*}}^{\prime },{e_{u}^{*}}^{\prime \prime }\right)/\tau )}{\sum_{v\in U}\exp\left(sim\left({e_{u}^{*}}^{\prime },{e_{v}^{*}}^{\prime \prime }\right)/\tau \right)},\\ L_{cl}^{UT}=\sum_{t\in T}-\log\frac{\exp(sim\left({e_{ut}^{*}}^{\prime },{e_{ut}^{*}}^{\prime \prime }\right)/\tau )}{\sum_{v\in T}\exp\left(sim\left({e_{ut}^{*}}^{\prime },{e_{uv}^{*}}^{\prime \prime }\right)/\tau \right)}, \end{array}$$

where sim(·,·) is the cosine similarity. τ is the temperature parameter. Moreover, for the item–tag interaction graph, we also compute the the contrastive loss of item side $L_{cl}^{I}$ and the contrastive loss of tag side $L_{cl}^{IT}$, formally,

(9) $$\begin{array}{c} L_{cl}^{I}=\sum_{i\in I}-\log\frac{\exp(sim\left({e_{i}^{*}}^{\prime },{e_{i}^{*}}^{\prime \prime }\right)/\tau )}{\sum_{v\in I}\exp\left(sim\left({e_{i}^{*}}^{\prime },{e_{v}^{*}}^{\prime \prime }\right)/\tau \right)},\\ L_{cl}^{IT}=\sum_{t\in T}-\log\frac{\exp(sim\left({e_{it}^{*}}^{\prime },{e_{it}^{*}}^{\prime \prime }\right)/\tau )}{\sum_{v\in T}\exp\left(sim\left({e_{it}^{*}}^{\prime },{e_{iv}^{*}}^{\prime \prime }\right)/\tau \right)}, \end{array}$$

Hence, the contrastive loss of CLPTR is defined as

(10) $$L_{cl}=L_{cl}^{U}+L_{cl}^{UT}+L_{cl}^{I}+L_{cl}^{IT}.$$

### 3.4. Objective Function

If the item *i* is annotated with the tag *t* by user *u*, we assume that user *u* prefers tag *t* over other tags $t^{\prime }\left(t^{\prime }\in T\setminus t\right)$, i.e., the partial relationship $t{>}_{u,i}t^{\prime }$ holds. We employ the BPR criterion [31] to learn model parameters. Supposing that all users and all partial relationships are independent, CLPTR optimizes the parameters of model by minimizing $L_{rec}$:

(11) $$\begin{array}{c} L_{rec}=\ln\prod_{(u,i,t)\in S}\prod_{t^{\prime }\in T\setminus t}P\left(t{>}_{u,i}t^{\prime }|\Theta \right)P\left(\Theta \right)\\ =-\sum_{(u,i,t)\in S}\sum_{t^{\prime }\in T\setminus t}\ln\sigma \left({\hat{p}}_{u,i,t}-{\hat{p}}_{u,i,t^{\prime }}\right)+\lambda_{\Theta }{\left||\Theta |\right|}_{F}^{2}, \end{array}$$

where $P(t{>}_{u,i}t^{\prime }|\Theta )$ denotes the probability that user *u* prefers tag *t* over tag $t^{\prime }$ when annotating item *i*. $\Theta =\{U,I,T_{U},T_{I}\}$ is the set of model parameters. Similar to traditional contrastive learning-based recommendation models, we jointly optimize the recommendation task $L_{rec}$ and the contrastive learning task $L_{cl}$ via the multi-task training strategy. Hence, the objective function of CLPTR is formalized as

(12) $$L=L_{rec}+\lambda_{cl}L_{cl},$$

where $\lambda_{cl}$ balances the contributions of contrastive loss for our proposed method.

## 4. Experiment

We conduct several groups of experiments on real-world datasets to evaluate the effectiveness of CLPTR via comparing against other state-of-the-art PTR models.

### 4.1. Datasets

We selected Last.fm and ML-10M (https://grouplens.org/datasets/hetrec-2011/, accessed on 1 September 2024) for experimental analysis. Specifically, the ML-10M dataset is an extension of the Movielens10M dataset, which was published by the GroupLeans research group. This dataset contains rating and tagging information assigned to movies by users. In addition, the Last.fm dataset is collected from the Last.fm online music system and contains social network, tagging and music listening information from the set of around 2 K users. Each user has a list of their most listened artists, tag assignments, i.e., tuples (user, artist, tag), and friend relations within the social network. Moreover, we remove users, items and tags that appear less than *p* times for all datasets. In our experiment, we set *p* to 5 or 10 and obtained four datasets, i.e., lastfm-5, lastfm-10, ml10m-5, and ml10m-10. Table 1 summarizes the statistics of all datasets.

### 4.2. Evaluation Metrics and Experimental Settings

Two widely used rank-oriented metrics, i.e., Precision@N and Recall@N, are chosen to evaluate all compared methods since PTR essentially is a ranking problem. For both metrics, we set N to 3, 5, and 10, respectively. In addition, we choose the following baselines for comparison:

- NGCF [32]: NGCF integrates GCN into personalized item recommendation models. In our experiment, we utilize user–tag interaction information as the input.
- PITF [6]: PITF models the three-order interactions among entities and utilizes BPR criteria to optimize the model parameters.
- NLTF [7]: NLTF utilizes Gaussian kernel to enhance the capacity of modeling the complex relations among entities.
- ABNT [8]: ABNT models the nonlinearity relationships among entities through multi-layer perceptron.
- GNN-PTR [16]: GNN-PTR integrates GCN into the PTR model and utilizes GCN to capture high-order collaborative signals among entities.
- LNGTR [17]: LNGTR utilizes the lightweight GCN to alleviate the training difficulty for GNN-PTR.
- GHPTR [33]: GHPTR explicitly injects higher-order relevance into entity representation through the message propagation and aggregation mechanism of GNN and leverages hyperbolic embedding to alleviate the problem of embedding distortion.

For all models, the learning rate η is selected from {0.001, 0.005, 0.01, 0.05, 0.1}, the embedding size *K* is set to 64, and the batch size is 1024. In addition, the regularization coefficient $\lambda_{\Theta }$ is tuned within {0, 0.00001, 0.0001, 0.001, 0.01}. For NGCF, GNN-PTR, LNGTR, CLPTR and GHPTR, the number of embedding propagation layers *L* is chosen from {1, 2, 3}. For GHPTR, the curvature is set to 1, and the target point is set to the origin of hyperbolic space. For CLPTR, the contrastive coefficient $\lambda_{cl}$ and noise coefficient ε both vary in {0.001, 0.01, 0.1, 0.2, 0.5, 1, 2, 5}. In addition, the Adam optimizer is utilized to optimize all model parameters. Each dataset is split into two parts, i.e., the training set $S_{train}$ and the test set $S_{test}$, with a ratio of 8:2.

### 4.3. Performance Analysis

Table 2 presents the results of performance comparison and the bold values indicate that the best performance of all comparison methods.

We have the following main observations:

- ABNT performs the worst on all datasets. One possible reason is that ABNT employs the multi-layer perceptron to capture the nonlinear relationships among entities, which introduces a large number of trainable parameters. However, with sparse interactions, ABNT is unable to accurately learn the embeddings of entities.
- NGCF performs better than ABNT. Although NGCF only models user–tag interaction information, NGCF utilizes GCN to extract high-order collaborative signals from the interaction behaviors, which enriches the embeddings of entities. This observation indicates that capturing high-order collaborative signals among entities is beneficial to PTR models.
- Compared to NGCF, NLTF achieves better performance. In fact, NLTF captures the third-order interaction among three entities rather than the second-order interaction.
- PITF generally outperforms NLTF. This indicates that explicitly modeling the pairwise interaction among entities is a promising approach for PTR systems.
- GNN-PTR is superior to PITF, because GNN-PTR utilizes the graph convolution module to effectively capture high-order collaborative signals among entities.
- The performance of LNGTR is better than that of GNN-PTR. This observation confirms that the cumbersome GCN may hinder the learning process of model parameters.
- Compared to LNGTR, GHPTR is better. One possible reason is that GHPTR utilizes hyperbolic distances to measure similarities between entities and leverages hyperbolic embedding to alleviate the problem of embedding distortion.
- On all datasets, our proposed PTR model achieves the best performance compared against all baselines. For instance, in terms of Pre@3 and Rec@3, CLPTR outperforms LNGTR by 11.6% and 16.15% on the ml10m-10. On the ml10m-5, the improvements over LNGTR are 7.5% and 7.7%, respectively. This observation demonstrates that integrating the contrastive learning module into the PTR model is helpful to accurately learn the embeddings of entities via capturing the invariances among the augmented views.

### 4.4. Ablation Analysis

In order to evaluate the contribution of each component in CLPTR, we conduct an ablation study. Our proposed method contains two important components, i.e., a graph convolution module and a contrastive learning module. If we remove the contrastive learning module, we term our proposed CLPTR as CLPTR-cl. In addition, if we drop both the contrastive learning module and graph convolution module, we term our proposed CLPTR as CLPTR-gcn. In fact, CLPTR-cl is equivalent to LNGTR and CLPTR-gcn is the same as PITF. The performance comparison analysis among CLPTR, CLPTR-cl, and CLPTR-gcn is presented in Table 3. In Table 3, the best performance is bolded.

From Table 3, we observe that each component is beneficial to the performance of CLPTR. Specifically, compared to the graph convolution module, the contrastive learning module has greater contributions to the performance of the PTR model. One possible reason is that the contrastive learning module helps the PTR model accurately learn the embeddings of entities via capturing the invariances among the augmented views.

### 4.5. Impact of Noise Combination

In this section, we discuss the impact of different noise combinations that generate augmented views on the performance of CLPTR. Specifically, ${\mathrm{CLPTR}}_{uu}$/${\mathrm{CLPTR}}_{gg}$ indicates that two augmentation operations utilize the same uniform noise/Gaussian noise to generate two augmented views. Moreover, ${\mathrm{CLPTR}}_{ug}$ represents that one augmented view is generated by injecting uniform noise and the other is created by adding Gaussian noise. Uniform noise and Gaussian noise are randomly sampled from uniform distribution U(0,1) and normal distribution N(0,1), respectively.

As shown in Table 4, the performance of ${\mathrm{CLPTR}}_{uu}$ is generally better than that of ${\mathrm{CLPTR}}_{ug}$, while ${\mathrm{CLPTR}}_{gg}$ performs the worst. This observation indicates that employing Gaussian noise to generate augmented views may harm the performance of PTR models. One possible reason is that the directions of the augmented vector of entities may be significantly changed since the noises sampled from Gaussian distribution N(0,1) could be negative numbers. Hence, the vectors augmented with negative elements may destroy the underlying semantics of the original view.

### 4.6. Parameter Sensitivity Analysis

#### 4.6.1. The Impact of τ

**Figure 4:**
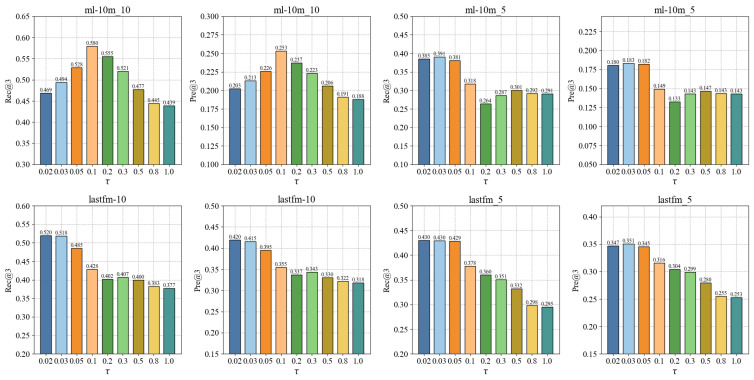
The impact of τ.

In this section, a group of experiments is conducted to analyze the sensitivity of τ. Figure 4 plots the performance changes of CLPTR with different values of τ. As shown in Figure 4, the performance of CLPTR is sensitive to the values of τ. In addition, when the value of τ is small, CLPTR shows competitive performance. For example, on ml10m-5 and lastfm-5, CLPTR achieves the best performance when τ=0.03. One possible reason is that the small value of τ makes CLPTR pay more attention to the differences among entities, and capturing individual differences is beneficial for the performance of CLPTR.

#### 4.6.2. The Impact of $\lambda_{cl}$

**Figure 5:**
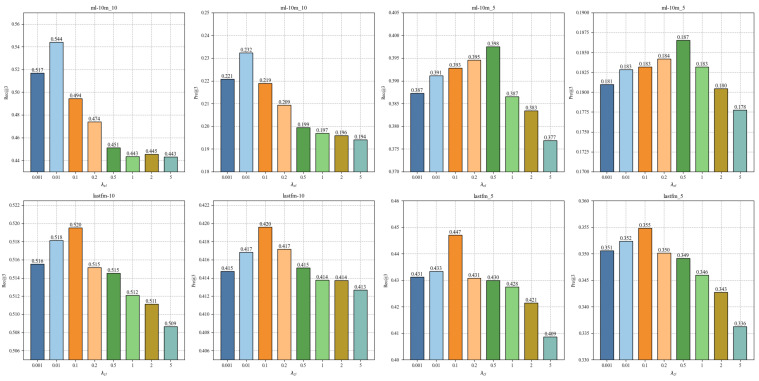
The impact of $\lambda_{cl}$.

We investigate how $\lambda_{cl}$ affects the performance of our proposed model. The contrastive learning coefficient $\lambda_{cl}$ is tuned within {0.001, 0.01, 0.1, 0.2, 0.5, 1, 2, 5} and other parameters remain unchanged. The results are presented in Figure 5. As shown in Figure 5, the performance of CLPTR gradually reaches its peak when $\lambda_{cl}=0.01$ on the ml10m-10, 0.5 on the ml10m-5, 0.1 on lastfm-10, and 0.1 on lastfm-5. Then, the performance of CLPTR begins to decline. This indicates that we should carefully tune the parameter $\lambda_{cl}$ for different datasets. In addition, a large value of $\lambda_{cl}$ makes CLPTR focus on the contrastive learning module and ignore the interaction patterns among entities, leading to suboptimal performance.

#### 4.6.3. The Impact of ε

**Figure 6:**
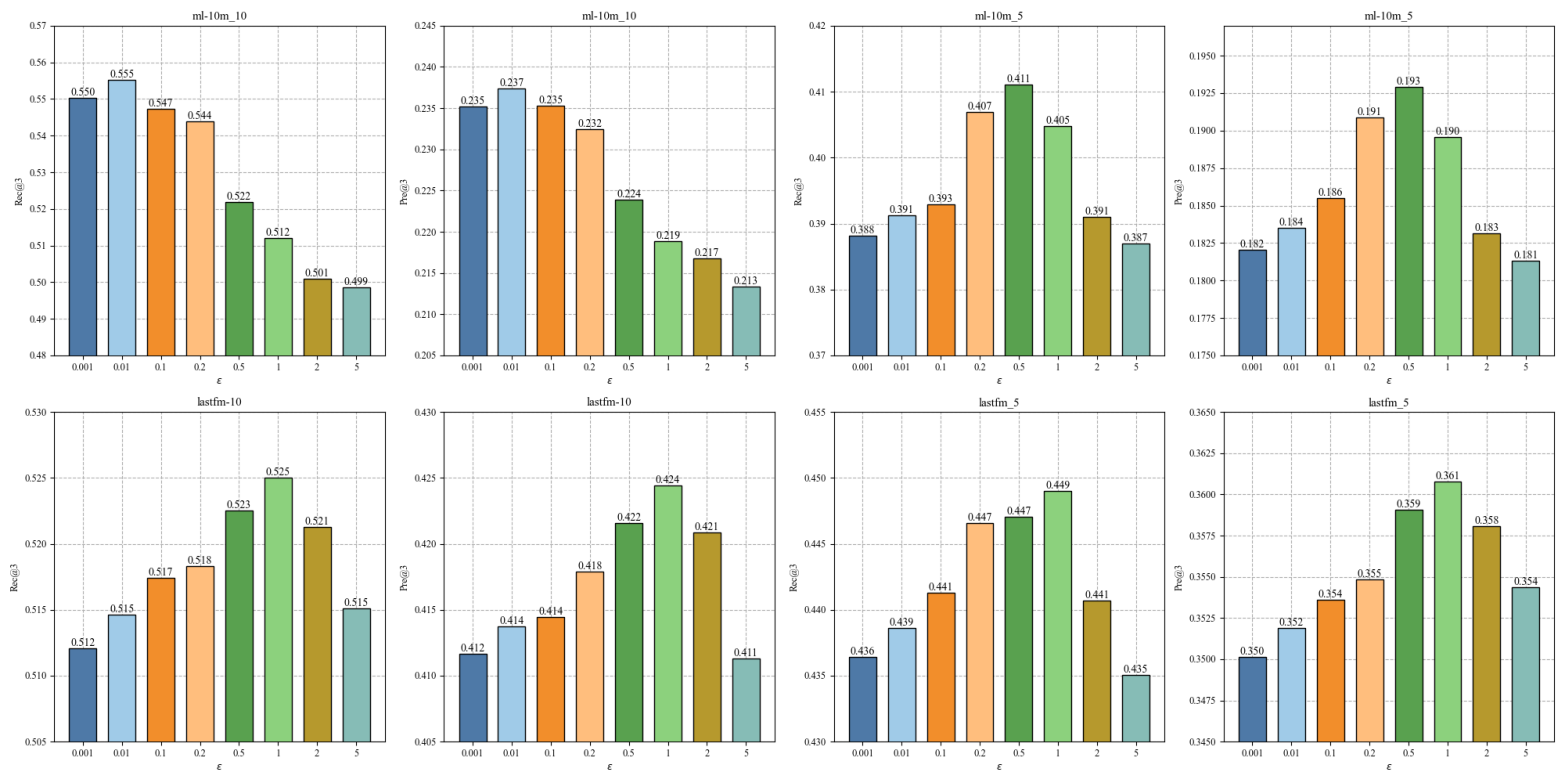
The impact of ε.

For the sensitivity of parameter ε, we change ε within {0.001, 0.01, 0.1, 0.2, 0.5, 1, 2, 5}, and other parameters remain unchanged. From Figure 6, when ε≤1, the performance of CLPTR continuously improves as we increase the value of ε on lastfm-10 and lastfm-5. Moreover, on ml10m-10 and ml10m-5, CLPTR achieves the best performance when ε is 0.01 and 0.5, respectively. When ε is large, the performance of CLPTR obviously degrades. This is because the similarities among the augmented vectors are completely dominated by noise vectors, making it impossible for CLPTR to extract the correct self-supervised signals.

## 5. Conclusions

In this paper, we propose the contrastive learning-based personalized tag recommendation algorithm, namely CLPTR. CLPTR generates augmented views by injecting noises into implicit feature representations, which not only greatly preserves the underlying semantics of the original interaction graphs but also avoids introducing the false negatives. In addition, we utilize the contrastive learning module to extract the self-supervision signals from user–tag and item–tag interaction graphs, resulting in accurately learning the representations of entities. We conduct extensive experiments on real-world datasets, and the experimental results demonstrate the superior performance of our proposed CLPTR compared with traditional personalized tag recommendation models.

## Figures and Tables

**Table 1:** Statistics of all datasets used in our experimental evaluation.

Dataset	#Users	#Items	#Tags	Interactions
lastfm-5	1348	6927	2132	162,047
lastfm-10	966	3870	1204	133,945
ml10m-5	990	3247	2566	61,688
ml10m-10	469	1524	1017	37,414

**Table 2:** Performance analysis.

Dataset	Metric	NGCF	PITF	NLTF	ABNT	GNN-PTR	LNGTR	GHPTR	CLPTR
ml10m-10	Pre@3	0.1244	0.1699	0.1436	0.0896	0.1933	0.2267	0.2507	**0.2530**
	Pre@5	0.0896	0.1173	0.1143	0.0759	0.1390	0.1748	0.1706	**0.1833**
	Pre@10	0.0571	0.0744	0.0714	0.0501	0.0842	0.1064	0.0960	**0.1106**
	Rec@3	0.3198	0.3770	0.3388	0.2210	0.4602	0.4990	0.5713	**0.5795**
	Rec@5	0.3837	0.4523	0.4334	0.3017	0.5461	0.6319	0.6343	**0.6653**
	Rec@10	0.4688	0.5205	0.5341	0.3858	0.6398	0.7698	0.6898	**0.7775**
ml10m-5	Pre@3	0.0950	0.1398	0.1323	0.0822	0.1455	0.1795	0.1915	**0.1929**
	Pre@5	0.0683	0.1021	0.0972	0.0628	0.1055	0.1388	0.1375	**0.1455**
	Pre@10	0.0438	0.0641	0.0596	0.0400	0.0672	0.0892	0.0797	**0.0908**
	Rec@3	0.2463	0.3208	0.2974	0.2089	0.3331	0.3819	0.4158	**0.4111**
	Rec@5	0.2820	0.3910	0.3560	0.2538	0.3965	0.4817	0.4799	**0.4992**
	Rec@10	0.3495	0.4623	0.4270	0.3039	0.4852	0.6008	0.5391	**0.6116**
lastfm-10	Pre@3	0.1739	0.2513	0.2443	0.1605	0.2647	0.3240	0.3382	**0.4244**
	Pre@5	0.1468	0.2088	0.2064	0.1367	0.2143	0.2652	0.2658	**0.3371**
	Pre@10	0.1140	0.1458	0.1249	0.0943	0.1462	0.1833	0.1772	**0.2168**
	Rec@3	0.2180	0.3204	0.2849	0.1579	0.3479	0.3949	0.4339	**0.5250**
	Rec@5	0.2878	0.4158	0.4017	0.2190	0.4529	0.5208	0.5367	**0.6587**
	Rec@10	0.4289	0.5654	0.5541	0.3034	0.5874	0.6830	0.6119	**0.7998**
lastfm-5	Pre@3	0.1679	0.2127	0.1949	0.1563	0.2324	0.2789	0.3043	**0.3591**
	Pre@5	0.1395	0.1789	0.1678	0.1353	0.1913	0.2325	0.2390	**0.2918**
	Pre@10	0.1023	0.1274	0.1191	0.1018	0.1327	0.1596	0.1547	**0.1911**
	Rec@3	0.2191	0.2571	0.2275	0.1569	0.3244	0.3415	0.3914	**0.4490**
	Rec@5	0.2907	0.3479	0.3239	0.2194	0.4170	0.4511	0.4776	**0.5736**
	Rec@10	0.4013	0.4814	0.4523	0.3298	0.5454	0.5861	0.5673	**0.6990**

**Table 3:** Ablation analysis.

Dataset	Metric	CLPTR-gcn	CLPTR-cl	CLPTR
ml10m-10	Pre@3	0.1699	0.2267	**0.2530**
	Pre@5	0.1173	0.1748	**0.1833**
	Rec@3	0.3770	0.4990	**0.5795**
	Rec@5	0.4523	0.6319	**0.6653**
ml10m-5	Pre@3	0.1398	0.1795	**0.1929**
	Pre@5	0.1021	0.1388	**0.1455**
	Rec@3	0.3208	0.3819	**0.4111**
	Rec@5	0.3910	0.4817	**0.4992**
lastfm-10	Pre@3	0.2513	0.3240	**0.4244**
	Pre@5	0.2088	0.2652	**0.3371**
	Rec@3	0.3204	0.3949	**0.5250**
	Rec@5	0.4158	0.5208	**0.6587**
lastfm-5	Pre@3	0.2127	0.2789	**0.3591**
	Pre@5	0.1789	0.2325	**0.2918**
	Rec@3	0.2571	0.3415	**0.4490**
	Rec@5	0.3479	0.4511	**0.5736**

**Table 4:** Performance comparison among different CLPTR variants.

Dataset	Metric	${\mathrm{CLPTR}}_{\mathrm{uu}}$	${\mathrm{CLPTR}}_{\mathrm{ug}}$	${\mathrm{CLPTR}}_{\mathrm{gg}}$
ml10m-10	Pre@3	0.2530	0.2431	0.2424
	Pre@5	0.1833	0.1774	0.1770
	Rec@3	0.5795	0.5678	0.5607
	Rec@5	0.6653	0.6696	0.6687
ml10m-5	Pre@3	0.1929	0.1323	0.1283
	Pre@5	0.1455	0.1085	0.1012
	Rec@3	0.4111	0.2687	0.2620
	Rec@5	0.4992	0.3597	0.3378
lastfm-10	Pre@3	0.4244	0.3647	0.3192
	Pre@5	0.3371	0.2969	0.2631
	Rec@3	0.5250	0.4277	0.3769
	Rec@5	0.6587	0.5654	0.4981
lastfm-5	Pre@3	0.3591	0.3244	0.2915
	Pre@5	0.2918	0.2690	0.2436
	Rec@3	0.4490	0.4088	0.3709
	Rec@5	0.5736	0.5335	0.4876

## Data Availability

The original data utilized in this paper are available at https://grouplens.org/datasets/hetrec-2011/, accessed on 1 September 2024. The prepossessed data and codes are available on GitHub: https://github.com/zar123123/CLPTR, accessed on 1 September 2024.
